# Evaluation of visual ergonomics in microsurgery: a real-time video processing solution

**DOI:** 10.1007/s00701-025-06694-2

**Published:** 2025-11-05

**Authors:** Gaukhar Mukash, Paavo Vartianen, Mastaneh Torkamani-Azar, Zeynel Karadis, Mehdi Faraz, Roman Bednarik, Pasi A. Karjalainen, Matti Iso-Mustajärvi, Ahmed Hussein

**Affiliations:** 1 Department of Technical Physics, Faculty of Science, Forestry and Technology, University of Eastern Finland, Kuopio, 70211 Finland; 2 A.I. Virtanen Institute for Molecular Sciences, Faculty of Health Sciences, University of Eastern Finland, Kuopio, 70211 Finland; 3 School of Computing, Faculty of Science, Forestry and Technology, University of Eastern Finland, Yliopistokatu 2, Joensuu, 80100 Finland; 4 Microsurgery Center of Eastern Finland, Kuopio University Hospital, Kuopio, 70210 Finland; 5 Faculty of Medicine, Assiut University, Assiut Governorate, 71515 Egypt; 6 Department of Computing, University of Turku, Turku, 20500 Finland

**Keywords:** Microsurgery, Image-guided surgery, Surgical microscope, Minimally invasive surgery, Instrument transparency

## Abstract

**Purpose:**

Surgeons’ visual and ergonomical challenges are long-standing concerns since the use of microscopes in surgical procedures. Although devices have been improved in the last few decades, the problem of narrow visual fields in high magnification surgeries persists. This study aims to identify the visual ergonomics challenges among microsurgeons and assess the need for novel assistive surgical solutions.

**Methods:**

The study consisted of two parts: a survey and a hands-on experiment. Sixteen surgeons from neurosurgery and otolaryngology (ENT) specialties were invited to semi-structured interviews on challenges encountered when using surgical microscopes and evaluation of the two proposed technological solutions: CPM (context-preserving magnification) and IT (instrument transparency). Following a demonstration of these software solutions, a survey utilizing a Likert scale was administered. The second part of the study involved twelve practicing neurosurgeons who performed a task using a novel solution and compared it to a standard operative microscope setting.

**Results:**

The most common challenges reported were visual obstruction of field by instruments (93.75%), blurring of structures and light reflection (81.25%), and loss of context (68.75%). 50% of surgeons agreed that adjusting zoom and focus takes a considerable amount of time from surgery and 56% stated that they had at least one episode of difficulty seeing depth. Notably, 69% of respondents expressed interest in testing prototypes of both proposed solutions in real surgery, with a particular preference for instrument transparency. Furthermore, the context-preserving magnification solution hands-on trial demonstrated a 40% reduction in task completion time for 60% of participants. However, one participant found no advantage, and others took longer to complete tasks with the solution compared to standard settings.

**Conclusion:**

Our solution addresses the top visual challenges and instrument obstruction remains a top challenge in high magnification microsurgery. We showed that surgeons are highly likely to use novel assistive technologies that provide wider visual field and transparent instruments.

## Introduction

Visualization is one half of the sensory ergonomics along with tactile perception in microsurgery [28]. Visual ergonomics in microsurgery is affected by several factors such as the type of device used and procedure [2]. MIS Microsurgery is often conducted using surgical microscopes, endoscopes, and exoscopes and recently with surgical robots. The use of surgical microscopes, the most common image guidance device in neurosurgery, imposes a range of visual challenges that can affect surgical performance. The most common ones are narrow fields of view, reduction of depth perception, instrument obstruction, and limited visual clues. Alongside ergonomic issues from handling the microscope, limited contact with tissue environment and sensitivity of structures make interruptions in workflow more harmful [14]. Surgeons engaged in microsurgery must therefore exhibit exceptional eye-hand coordination, heightened dexterity, and increased cognitive workload to manage these challenges effectively [2, 25]. Some of these requirements can be approached by training fine motor skills and by built-in features such as zooming and focusing [5, 8].

Optical zooming in the field of view reduces contextual awareness which makes it difficult for surgeons to identify nearby structures. The presence of multiple instruments within the operating field further obstructs the view from the already limited visual field [15]. Current surgical microscopes require manual adjustments for focus, magnification, and positioning, which can interrupt the surgical flow and consume time during the surgery [12]. Moreover, there are other visual challenges during microsurgery that cannot be fully addressed by a microscope or by other devices alone. For instance, high magnification can affect depth perception (stereopsis) so that the distance between different tissues becomes distorted. Secondly, frequent switch between different zoom levels requires additional cognitive work from the surgeons to adjust to the new surgical view potentially leading to increased error rates [4, 6, 11]. These challenges emphasize the need for advanced visualization solutions that can enhance surgical precision and efficiency.

**Fig. 1:**
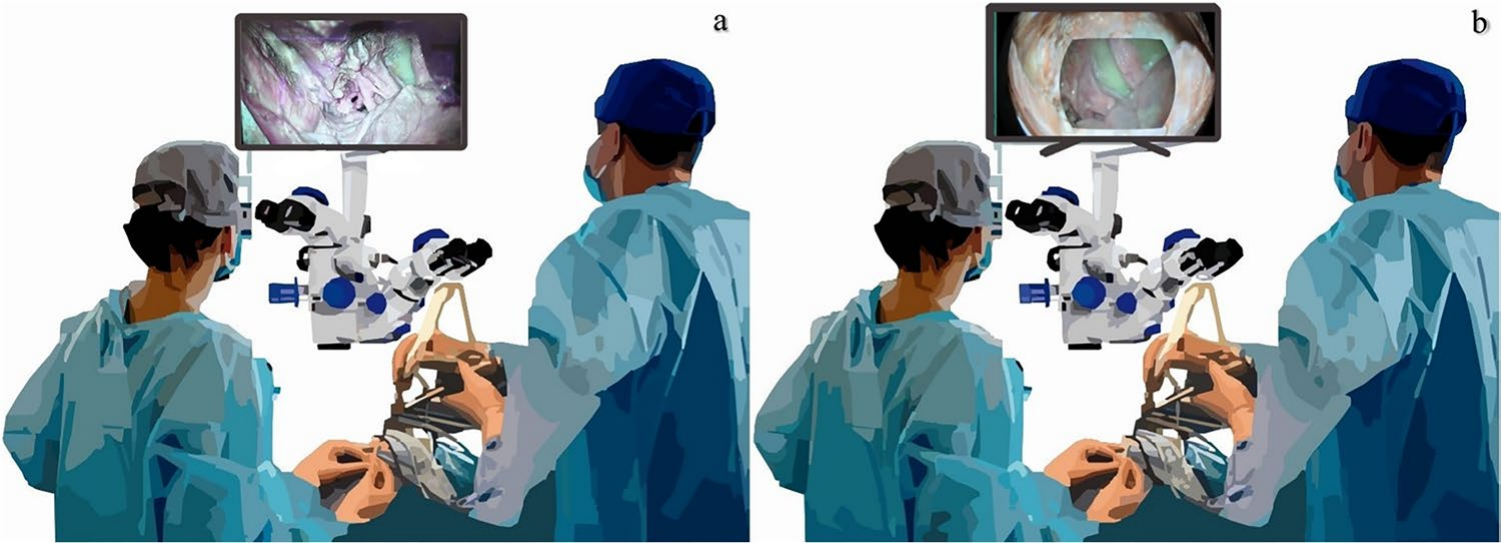
Conceptual comparison of the conventional visualization and the proposed solution in neurosurgery. **a** A neurosurgeon uses a microscopic-assisted endoscopic setup requiring frequent alternation between the microscopic eyepiece and the endoscopic display. **b** A neurosurgeon utilizing image processing-based visualization solution, where image from both microscope and endoscope is processed in real time into a single, non-overlapping, view on one display

The current study aims to develop a visual solution that address challenges of visual ergonomics in microsurgeries. First, we identify the key visual ergonomic issues encountered by neurosurgeons using structured questionnaire and interview. Next, based on these insights we develop and evaluate the visual solutions that are aimed at addressing these challenges. The first feature of our solution combines images from two cameras to produce a single expanded view based on the projective transformation techniques. The second feature involves instrument transparency/subtraction from the operating field. Our solution is applicable for surgical microscopes and relies on the multi-camera setup that provides complementary views (Fig. 1). Our hypotheses are (1) visual ergonomics issues are perceived as relevant and frequently encountered during microsurgery and (2) proposed solutions particularly context preservation expansion can improve task performance and reduce workflow interruptions compared to standard microscope settings.

## Materials and methods

This study was conducted in two sequential parts: study part 1 and study part 2. The study part 1 was conducted first, in winter-spring 2024 and earlier in the development process with most participants being from Finland. This study focused on the assessment of visual ergonomics challenges and users’ interests and involved a survey with a video demonstration aimed at assessing the commonly encountered visual ergonomics challenges. In addition, we showed the in-development videos of our proposed features to neurosurgeons to receive their professional evaluation of the feasibility and usability of these features in improving the visual ergonomic challenges in real-world settings. In the study part 2, we focused on user feedback and performance and conducted a hands-on evaluation of proposed solutions using simulated surgical tasks in comparison’ with conventional microscope setup. This approach was used to capture neurosurgeons’ experience and direct the development of features. Study part 2 was carried out later in fall 2024 when the working prototype was available.

### Study part 1: survey with video demonstration

The cohort of interest includes practicing neurosurgeons, neurosurgery residents, and medical students who utilize surgical microscopes for training and surgical practice.

***Participant selection.*** The study subjects were recruited at the Microsurgery Center of Eastern Finland, Kuopio University Hospital’s site and online by Webropol, the University of Eastern Finland (UEF) online survey tool. Participants were selected based on their surgical specialty, experience working with surgical microscopes, and performing microsurgeries. Surgical residents of all years of study were also invited if they had some experience working with surgical microscopes. Surgeons of other specialties that do not involve microscopic surgery and medical students were excluded. Participants were recruited from January 2024 to May 2024.

***Ethics statement*.** This study was conducted after approval by the UEF Committee on Research Ethics in accordance with the Finnish Medical Research Act (1999/488). All participants were instructed on personal data use and provided informed consent.

***Survey design*.** The survey consisted of two parts: (a) A semi-structured interview and (b) a written questionnaire. Interview questions were designed in three sections: demographics, current challenges, and alternatives (proposed solutions) (Table 1). The demographic data included hospital units, position, and professional experience in years. The questions on current challenges aimed at identifying visual ergonomics difficulties; examples or open-ended explanations (guidance Table 1) were provided by the interviewer whenever possible. In the last section titled as alternatives, the use of other minimally invasive surgery (MIS) devices such as endoscopes was also assessed.

**Table 1:** Semi-structured interview questions and guidance content

Sections	Questions
Demographic data	Hospital unit, position, and experience in years
Challenges	What kind of surgeries do you perform on a regular basis using surgical microscopes?^a^
	What are the most common challenges that you experience when using surgical microscopes?
	How often do you use built-in features of surgical microscopes like zooming in/out, focus adjustments, lightning, etc.?
	Do you think adjusting these features consumes time?
	Do you experience visual fatigue often?^b^
	Have you ever had trouble with depth perception during surgery? If so, can you describe what happened and how it affected your performance?
Alternatives	Have you ever needed to use endoscopic-assisted microscopy? How much time is needed to set it up?^c^
	Can you name any benefits for setups used in endoscopic-assisted microscopy?

^a^ Type of surgery (vascular, skull base) and % of surgeries that require microscope use

^b^ Related to device use

^c^ If exoscope was used before, the participants were asked about its benefits

Study participants were interviewed face-to-face or online in the presence of one or two team members on their experience with surgical microscopes (measured in years), common type of surgeries they perform and challenges they encounter. As the interview was semi-structured, participants were free to share their opinions during the conversation's natural flow.

**Fig. 2:**
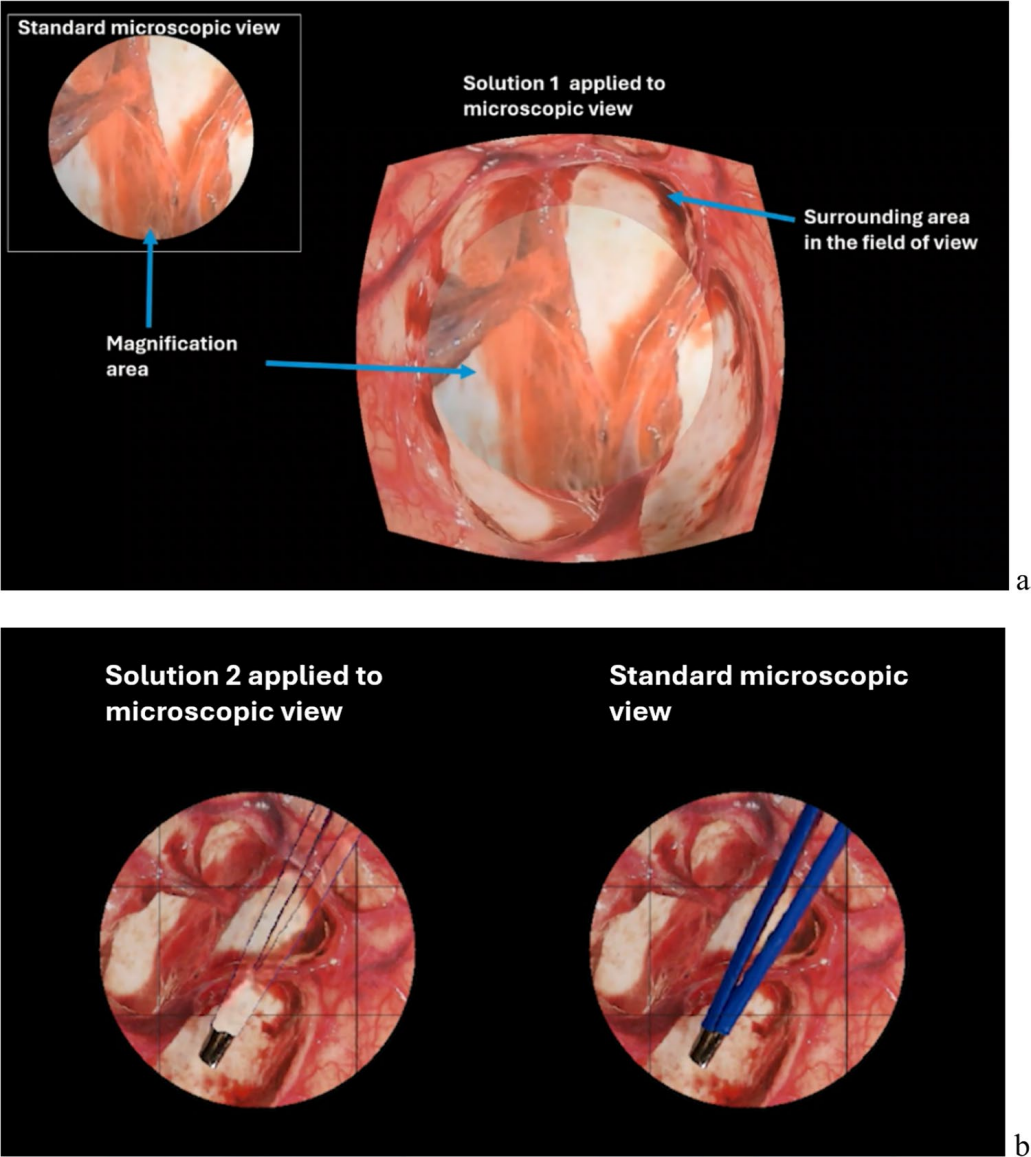
Screenshots from Solution 1 (context-preserving magnification or CPM) and Solution 2 (instrument transparency or IT) videos. **a** Solution 1 encompasses two fields of vision on a single microscopic view. The central area shows a magnified area of interest, which represents the surgeons’ performing area while the surrounding area appears in the field of view at a lower magnification level. Which provides an additional visual information area. **b** IT video shows the side-to-side comparison of standard appearance of surgical instrument and semi-transparent version of it

The second part of the survey, a questionnaire, consisted of open-ended and multiple-choice questions that helped to quantify the visual ergonomic difficulties followed by Likert scale questions on the proposed solutions that were played back for participants using two demonstration videos (Fig. 2, Table 2). The 5-point Likert scale questions were structured to evaluate participants’ perceived usability and possibility to use proposed solutions numerically. They also allow us to assess the neurosurgeons’ perceived usability and potential advantages of the two solutions in real surgeries. The questionnaire was divided into four parts based on the content, namely, Challenges, context-preserving magnification (CPM), instrument transparency (IT), and application.

**Table 2:** Questionnaire content. CPM: Context-preserving magnification. IT: Instrument transparency

Sections		Questions	
Challenges		Q1. What kind of challenges have you experienced during microscopic surgeries?	
		Q2. Does changing the zoom level interrupt the workflow?	
		Q3. How is the zoom control in the microscope you often use?	
		Q4. How much time in seconds does it take to change the zoom level?	
Solution 1 (Context-preserving magnification, CPM) & Solution 2 (Instrument transparency, IT)			
Q1	How much would you like to try the prototype of Solution 1/Solution2?		
Q2	Did you find Solution 1 to be helpful for identifying more anatomical landmarks?		How do you expect the effectiveness of Solution 2 on your visual ergonomics of the surgical scene?
Q3	Did Solution 1 cause any disorientations?		Do you find Solution 2 presented any artifacts or visual noise?
Q4	Would you be comfortable using Solution 1 in a real surgical setting?		
Q5	Do you think Solution 1 could help in saving surgery time in avoiding the frequent exchange between different zoom levels?		Do you think that using solution 2 would improve your performance as a surgeon?
Q6	Do you think that using Solution 1 would improve your performance as a surgeon?		
Application		How do you think these solutions are helpful in demonstrating the surgical field on the operating room (OR) screens for other OR personnel?	
		How do you think these solutions are helpful in demonstrating the surgical field in the video management systems and surgical video recordings for educational purposes?	
		In what ways do you think the proposed surgical software solutions could improve your surgical outcomes?	
		Would you recommend the proposed surgical software solutions to other surgeons?	

***Demonstration videos*.** In the CPM and IT sections of the questionnaire, participants were shown short video demonstrations of the solutions (Fig. 2a and b). Solution 1 demonstration included a 28-s-long video clip composed of two geometrically aligned videos of a brain tissue (standard microscopic view and CPM applied to microscopic view). The aim of CPM was to combine the video outputs from a microscope and a secondary camera into one output. The area of interest was magnified to a greater extent compared to the surrounding area, thus allowing the operating surgeons to avoid changing zoom levels to explore surrounding areas (Fig. 2a). Instrument transparency demonstration included a 50-s-long video of two standard microscopic views of brain tissue in which a pair of forceps were placed over the tooth tissue (Fig. 2b). The tissue forceps were moved over the brain tissue twice and locked twice. The left microscopic view shows an application of IT which makes the body of the instrument, but not its tips and outline edges, transparent. This is in contrast with the standard microscope view of the same scene on the right.

### Study part 2: the prototype and hands-on experiment

**Fig. 3:**
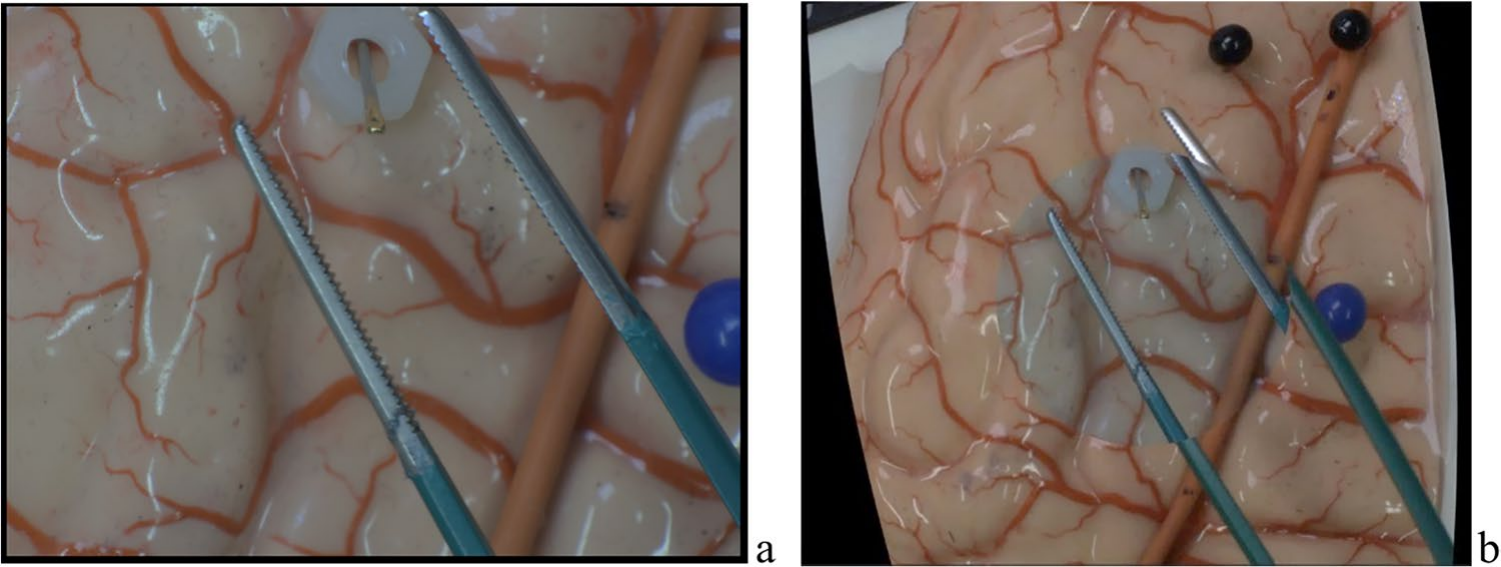
Screenshots from the prototype test task. **a** Single camera output video where a zoomed section of the temporal cortex is visible. A metal pin is placed in the center of the field and a ring is placed on the outer corner of field. Zooming out and focusing are needed to identify the ring location at the beginning of the task. **b** Two camera output videos where a whole temporal cortes of brain is visible

***Prototype setup*.** The hardware system consists of two USB 3.0 cameras (Ximea, Germany) of resolution 3.1 Mpix, the first camera equipped with a zoom lens (focal length range:18—108 mm) acts as a microscope. The second camera equipped with a fixed focal length lens (12.5 mm) is used by a proprietary algorithm to expand the field of view (Fig. 3). The cameras are connected to USB 3 ports of a laptop PC. The proprietary algorithm runs on the Microsoft Visual studio IDE, utilizing CUDA computing in Windows 11. Essential hardware specifications of the laptop are; CPU: Intel Core (TM) i7-11850H, RAM: 32 GB, and GPU: Nvidia RTX 3070 mobile. The algorithm utilizes both CPU and GPU processing of the laptop. Details of the algorithm and its implementation are proprietary to the project team. In This study focuses on the usability and feasibility testing from the perspective of neurosurgeons as the primary users. Especially, to enable visualization of the expanded view on a screen, while maintaining size of the microscope view large enough. A mathematical algorithm to deform the image of the secondary camera was developed. The frame rate and time delay of the video processing were tested by the team members, and by surgeons, who have experience in using digital microscopes and endoscopes in surgeries, to get subjective feedback.

***Hands-on prototype testing*.** The test was conducted in a training environment at the World Federation of Neurosurgical Societies (WFNS) educational day at the 14th Asian Congress of Neurological Surgeons, Cairo, between 18 to 20th of December 2024. Twelve neurosurgeons were asked to perform a task on a brain silicone model (TemporalBox, UpSurgeOn Srl, Italy). The task consisted of placing a ring on top of a pin using toothed forceps and participants were free to use either one or both hands, according to their preference (Fig. 3). First, participants were asked to perform the task looking at a display that demonstrated output from a single camera, mimicking conventional microscopic view (Fig. 3a). Instructions on how to adjust camera setups including the zoom and focus controls were explained beforehand. Next, participants were asked to perform the same task using our CPM (Fig. 3b). Time taken to perform the task, workflow interruptions, and task outcome which is placing a ring on top of a pin were measured in both settings. Workflow interruptions were defined as conditions when a participant removes a hand from the surgical field to adjust camera setup. If hands were removed for other reasons than microscope related such as handling the instrument it was not considered as workflow interruption. It is important to reiterate that participants were different between study part 1 (questionnaires and demos) and study part 2 (task conductance).

***Data analysis*.** The primary research methods of this study were interviews and questionnaires. The demographic data was demonstrated as percentages and means. Qualitative data was grouped based on the frequency of similar responses. Multiple-choice questions and Likert scale questions were analyzed using SPSS. Time taken to complete the tasks was compared between two settings using the paired t-test and workflow interruptions (ordinal data) was compared using Wilcoxon matched-pairs test due to small sample size. The Cronbach’s alpha method was used to assess multiple choice questions (MCQ) reliability for sections of Solution 1 (context-preserving magnification) and Solution 2 (instrument transparency). All figures were drawn on GraphPad Prism 10 software.

## Results

*Demographics***.** Sixteen surgeons from four countries (Finland, Scotland, Germany, and UK) were recruited between the period of 29th of January to 17th of May 2024. Most surgeons (94%) were practicing neurosurgeons or completing residence in neurosurgery working at Neurosurgery unit and only one ENT (ear-nose-throat) surgeon was recruited (Table 3). The average length of experience in microsurgery operations was 15.6 years. The variety of surgeries ranged from spinal surgeries to skull base surgeries. The ratio for use of microscope in operated surgeries varied from 60 to 100%, although the estimation was subjective and best on the participants’ deduction.

**Table 3:** Participant demographics (*N* = 16)

Variable	Number	%
**Hospital unit**		
Neurosurgery	15	94
ENT surgery	1	6
**Position**		
Head of unit	2	12.5
Chief surgeon	2	12.5
Assistant	8	50
Resident	4	25
**Experience in neurosurgery**		
< 5 years	4	25
5–10 years	5	31.25
10–20 years	3	18.75
20 + years	5	24

**Fig. 4:**
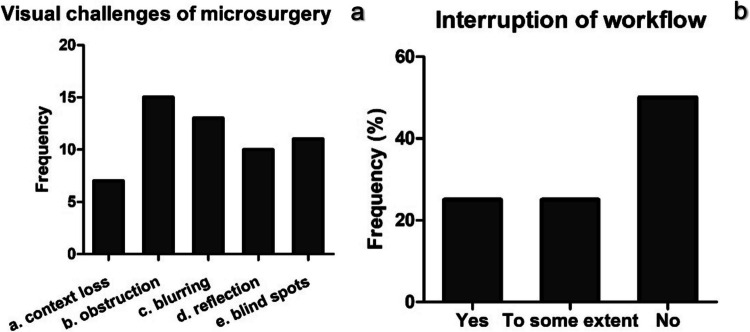
Responses to Q1 and Q2 of the section on challenges from the questionnaire of the semi-structured interviews (*N* = 16). **a** Q1 answer options a. Loss of context with high magnification, b. Visual obstruction by surgical instruments, c. Blurring of some structures due to limited depth of field, d. Blurring of surgical instruments and light reflection, e. Loss of certain areas from visual field (surgical blind spots) (**b**) Workflow interruption during microscope use

*Visual ergonomics challenges***.** In response to the interview questions, the most common type of visual challenge in microsurgery was the visual obstruction by surgical instruments followed by blurring of some structures due to limited depth of field (Fig. 4a). Loss of context with high magnification constituted the least common challenge as reported by 7/16 surgeons. Other visual challenges reported in a free-form comment were loss of orientation in the operating field, insufficient lighting, narrow corridor of view, lack of 3D vision, depth identification and loss of instrument shaft from the view.

All participants stated that they use the zoom, focus, and light adjustment features of surgical microscopes all the time. 25% of participants reported that adjusting these features consumes a considerable amount of time from surgery (Fig. 4b). Another 25% agreed that to some extent it takes time from surgery, while half of the participants answered negatively.

**Fig. 5:**
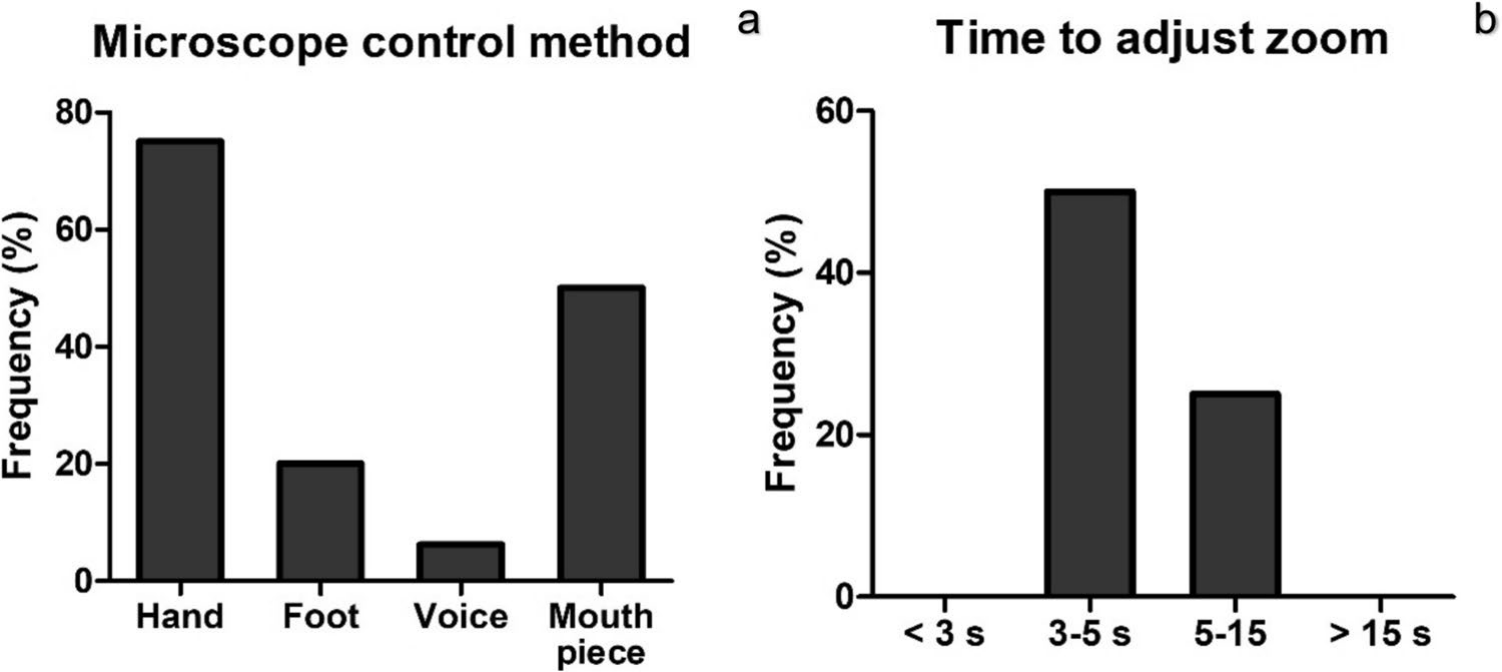
Microscope control method by surgeons (**a**) and time taken to adjust zoom level in seconds (**b**) from semi-structured interviews (*N* = 16)

As each microscope has different control settings, participants were asked on the method of zoom control and how long it takes to adjust it in their practice (Fig. 5). Hand control was the most common (75%) setup for adjusting the zoom level followed by mouthpiece (50%), foot control (20%) and voice control (6.25%) (Fig. 5A). It takes about 3–5 s to adjust zoom level for the majority of surgeons while 25% of respondents spend 5–15 s for zoom adjustment (Fig. 5B). Interestingly, none of the participants takes less than 3 s or more than 15 s for zoom accommodation.

**Fig. 6:**
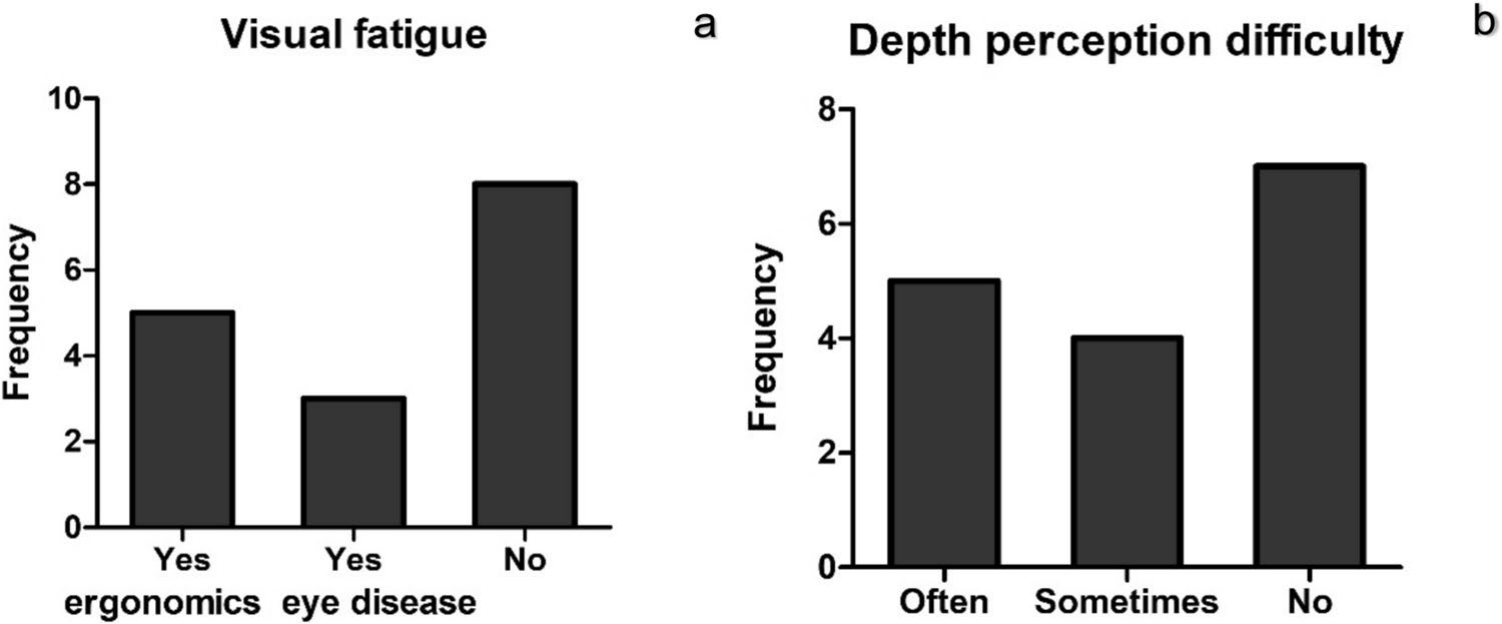
Visual fatigue (**a**) and depth perception (stereopsis) difficulty (**b**) during high magnification microsurgery from semi-structured interviews (*N* = 16)

Visual fatigue was common for half of the participants, but only five of them mentioned its association with ergonomic factors in their practice while the rest experienced it due to pre-existing eye diseases such as myopia and hyperopia (Fig. 6a). Depth perception in high magnification surgeries was commonly reported by nine surgeons while the rest of respondents answered that they do not encounter such problem (Fig. 6b). Among free-form answers, it was noted that some of the surgeons adapt to uncomfortable body positions for better visualization. One of the respondents admitted that depth perception problem persists for 2–3 min after changing positions from the main view to the assistant view.

*Alternatives*. In the next set of questions, we assessed participants’ views on the use of alternative devices such as the endoscope. For that reason, participants were asked about their experience of using endoscopes and benefits of endoscope-assisted microscopic neurosurgery in a free form. Only four participants had ever used endoscope-assisted microscopic setup, and their experience was limited to one-two procedures per year. Two surgeons had received training on surgical models with endoscope but had not used it during real operations. The main advantage of endoscope assisted microsurgery was the better visualization of narrow and deep fields of view compared to microscope used alone; however, it was also noted that the endoscope size may not be suitable for skull base surgeries. In addition, four surgeons had used exoscope and two of them stated the advantages of exoscope over microscope in terms of manoeuvring flexibility and biopsy sampling. Two other surgeons did not consider the exoscope superior and instead noted that its use requires adaptation.

**Fig. 7:**
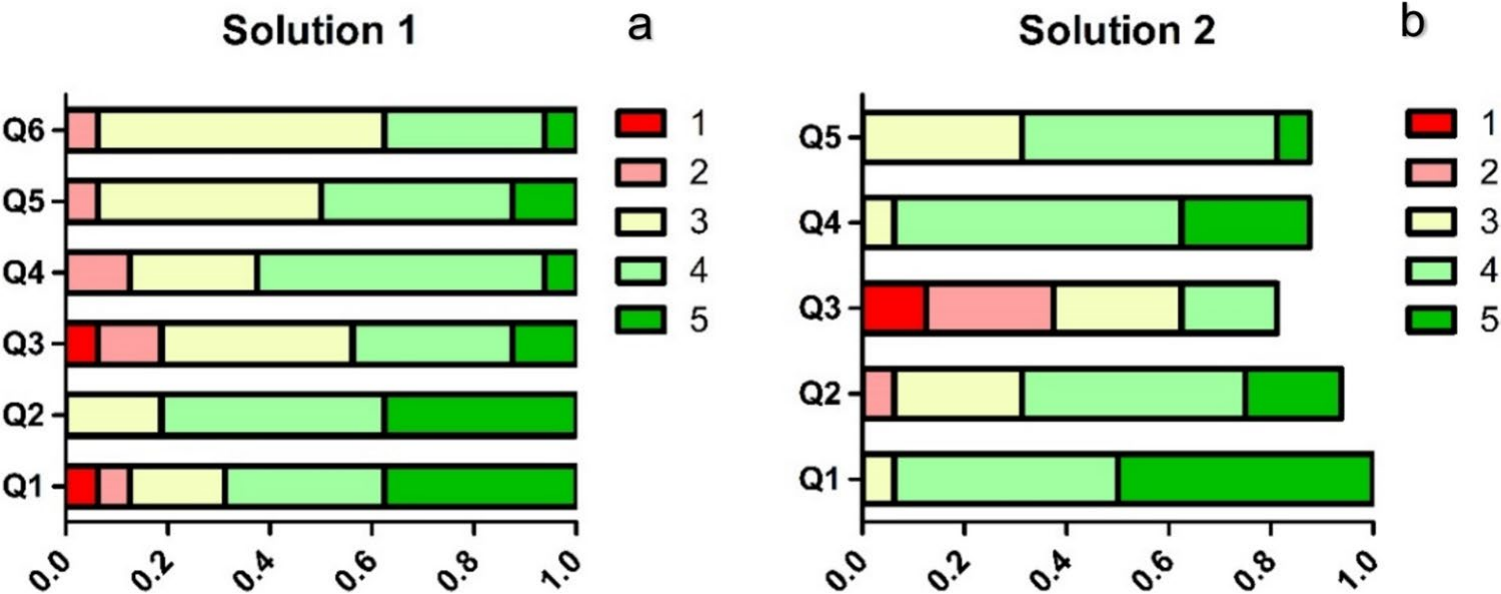
Results of Likert-scale evaluations for Solution 1 (a, context-preserving magnification or CPM) and Solution 2 (b, instrument transparency or IT), *N* = 16. Questions are provided on Table 2. Scores from 1 to 5 given based on the level of agreement: strongly agree – score 5, strongly disagree – 1. Scoring is reversed for Q3 (‘Did Solution 1 cause any disorientations?’ and ‘Do you find Solution 2 presented any artifacts or visual noise?’) for both solutions as the question addresses disadvantages, e.g. strongly agree – 1, strongly disagree – 5. Each score’s proportion is given in different shades of grey. The lighter the shade the higher the agreement level. Some of the respondents did not answer to MCQ of Solution 2, therefore proportion for Q2-Q5 in part b is below 1.0

*Proposed Solutions***.** 69% of respondents agreed that they would like to try the prototypes of both solutions (Fig. 7). More than half of the respondents stated that they would like to try Solution 1 (context preserving magnification) in a real surgical setting while the percentage was 81% for Solution 2 (instrument transparency). 81% of surgeons positively evaluated assistance of deep learning-based context-preserving magnification to be helpful for identifying more anatomical landmarks (Fig. 7a, Q2). The potential of Solution 1 (context preservation) to decrease the operating time and improve surgeon’s performance was supported by over 35% of participants. However, some surgeons (44%) stated that seeing two different levels of zoom can cause disorientation and can take time to adapt. Over 60% of respondents agreed that instrument transparency in Solution 2 can be effective in improving visual ergonomics during high magnification surgeries (Fig. 7b). Although one fifth of surgeons stated that Solution 2 can cause visual artifacts, more than half agreed that it can improve their surgical performance. The internal consistency of the questionnaire was high for CPM (α = 0.92) and lower for IT (α = 0.48).

*Application potentials***.** In the last section of the questionnaire presented in Table 2, we assessed the potential application of the proposed solutions in real surgeries in the OR and educational settings. Surgeons (n = 5) noted that the context preserving magnification solution with two fields of view can be helpful in improving the comprehension of anatomy and use of microsurgery instruments. The instrument transparency feature was evaluated as a practical solution to overcome the constant blockage caused by instruments in use within the surgical field. Participants indicated the instrument transparency solution would provide a better teaching experience for other personnel involved in the operation and even medical students or residents shadowing or observing the surgery. Surgeons also proposed that IT could be help prevent or reduce or the risk of injuries to vital physiological structures caused by compression. Regarding IT, bleeding control was seen as main usage. Although, some of the surgeons agreed that proposed solutions are advantageous only to the surgeons, 11 participants stated that they will recommend it to other surgeons.

**Fig. 8:**
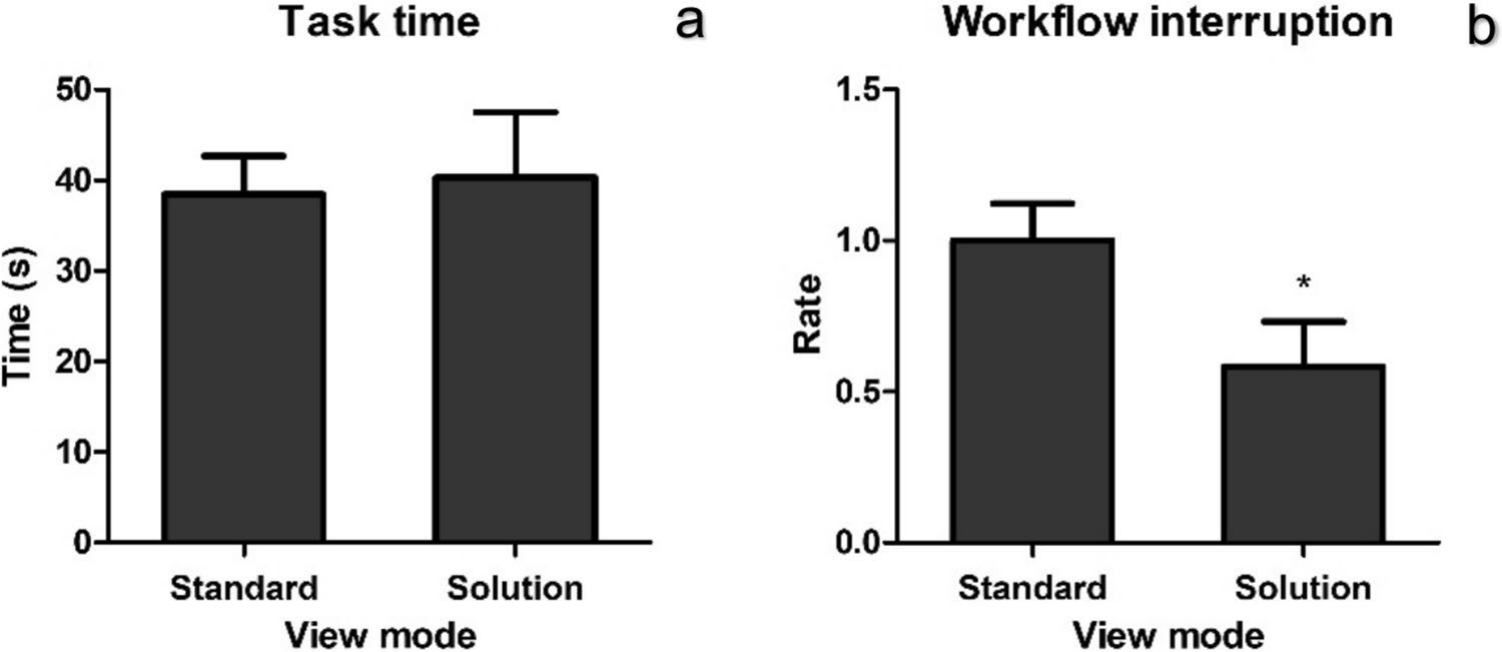
Time (**a**) and workflow interruption measurements (**b**) during prototype testing, *N* = 12

*Prototype testing***.** 58% of participants spent 40% less time completing the task using Solution 1 (context preserving magnification) compared to standard setting (Fig. 8A). The standard setting implies the same setup but only one video output from a single camera. However, the overall results indicate that there is no significant difference in the time taken to complete the task between Solution 1 and standard setting use (*p* > 0.05). On the other hand, there was significant reduction in workflow interruptions (*p* = 0.02). Notably, CPM had an advantage of eliminating microscope adjustments for this task which prevented interruption of workflow. Additionally, for one participant, there was no difference in task completion time, and four participants spent more time longer to complete the task using the CPM. Participants who found CPM beneficial in terms of saving time also noted that CPM is useful when a larger object is out of view and would require significant zoom out even though the view can be defocused. All participants were able to accomplish the task goal of placing the ring.

## Discussion

The study aimed to identify visual ergonomics challenges during microsurgery and assess the need and advantage of novel software solution compared to current microscopic features. Interview results showed that the most common type of visual challenge during high magnification surgery is obstruction by surgical instruments (94%) followed by blurring of some structures due to limited depth of field (81%). Other issues encountered include disparity in depth perception and narrow field of view. Most common microscope related reported issues were workflow interruption by adjusting zoom and focus (50%) and depth misperception (56%). Other difficulties reported in the free form were of ergonomical origin such as uncomfortable body position and muscular pain. While novel solutions tested by neurosurgeons have shown to decrease the task completion rate compared to the conventional microscope, they did not result in a significant reduction in workflow interruptions.

Visual ergonomics is a fundamental concern across various fields of surgery including ophthalmology, otolaryngology and reconstructive surgery [26]. In neurosurgery, surgeons often face prolonged positions commonly during high precision anastomosis [30]. These ergonomic challenges are closely related to visual challenges of working with microscopes and are often compromised to having a better view of surgical area [14]. Addressing visual ergonomics can significantly mitigate musculoskeletal disorders associated with neurosurgical practice improving surgeons’s performance and patient output [16, 27]. Our study reinforces these findings and quantifies the prevalence of visual ergonomics challenges in neurosurgery.

Recently adjustments to microscopes have been improved by technical advancements, such as mouthpieces and voice control. However, these innovations are not widely used due to usability issues [12]. Depth perception (stereopsis) is an important skill in microsurgery and essential for training in image guided surgery. Impaired depth perception is associated with decrease in surgical performance and learning curve [7]. Although stereopsis can differ significantly between individuals, microsurgery skills can be enhanced by technology assisted learning [10]. Exoscopes and endoscopes were developed to address 3D environment visualization and specialized hand–eye coordination with both having own advantages and disadvantages. Despite recent advancement endoscopic modalities still suffer from lower imaging resolution while exoscopes still remain inferior in depth perception to standard microscopes [3, 22]. Here we showed that depth discrimination is still a common problem in microscopic surgery which is not addressed widely.

Both exoscope and endoscope in a combination with microscope act as secondary cameras to provide view from different perspective. Endoscope assisted microsurgery (EAMS) is a promising method in certain pediatric procedures, but the issue of operating field obstruction by instruments and visual workload increases [9, 20]. In addition, visual output of these instruments is displayed on a separate screen which requires surgeons to alternate between microscope’s ocular and screen. Here, we developed an algorithm that combines visual outputs from microscope and a secondary camera into one. The idea of context-preserving magnification and instrument transparency have been largely supported by surgeons (Fig. 7). Our solution addresses the issues of instrument obstruction, loss of context and depth perception to some extent. Furthermore, the need of introducing a new display is eliminated and output is observed in real-time.

Current developments aimed at addressing visual ergonomics challenges across various microsurgical specialties are presented in Table 4. Many of these technologies are in the early stage of development and highlight the relevance of proposed solutions with different approaches. Below we describe key features of current developments are compare them with our own solutions.

**Table 4:** Comparison of current developments on visual obstruction during microsurgery

	CPM & IT	CARET by Okubo et al. [21, 24]	NOTES by Lerotic et al. [17]	Visual compensation system by Koreeda et al. [13]
Instruments	Microscope and 2nd camera	Laparoscope	Endoscope	Endoscope
Number of cameras	2	2–3	1–2?	2
Real-time processing	Yes	Yes	No	Yes
Processing time	20 ms	111 ms	NA	62.4 ms
Instrument transparency	Yes	No	No	Yes
Context-preserving magnification	Yes	No	Yes	No

Miranda-Luna et al. proposed construction of the panoramic view of bladder neoplasm endoscopic images, but it is applicable only for static images. It requires image analysis after the procedure and cannot affect the decision-making during the intervention [19]. Lerotic et al. presented a method that utilizes parallax correction scheme that extends the visual field during Natural Orifice Transluminal Endoscopic Surgery (NOTES). It gives advantage in viewing peripheral tissue without disturbing the focal view, but it has the same limitation as the previous study, only static images can be obtained [17]. The real-time field expansion is challenging in microsurgeries as usually only one camera is used e.g. endoscope or microscope. The introduction of the second camera allows to obtain video from another angle. This method was utilized by Okubo et al. [21, 24] who developed camera-retracted trocar (CARET). CARET is a conventional trocar with a built-in tiny camera at the tip that can be retracted during insertion and expanded inside the abdominal cavity. Simultaneous usage of two CARETs during laparoscopic surgery produced panoramic image by mosaicking and tracking methods. Benefits of this method are that it can produce images even in the case of 0% overlap and processing speed is 9fps faster than the conventional mosaicking method. However, tracking accuracy is still low. In 2021, the method was further improved using Oriented Robust Binary Simultaneous Localization and Mapping (ORB-SLAM). The new approach has higher accuracy and speed (21 frames per second (fps)) as it does not depend on image overlap [1].

Instrument transparency, which is one of our solution features, has been developed using the endoscope by Koreeda et al. They utilized image transformation and segmentation to combine video outputs from two endoscopes into one [13]. Like our solution, the tip and outline of the instrument remain visible while the shaft is semitransparent. However, the experimental setup consisted of fixed endoscopes whereas in real surgical setting this is seldom the case.

Overall, these emerging solutions indicate a need for addressing visual ergonomics in microsurgery and the early approach methods. Our work aims to contribute to the issue in the neurosurgery field and overcome limitations encountered by other developments focusing on feedback from neurosurgeons.

The current limitation of our solution remains the placement of the second camera adjacent to the microscope. Although the camera can be in a fixed position relative to microscope, the narrow corridor of the operating field can block the view of the secondary camera. This limitation is under evaluation by employing EAMS setup where the endoscope acts as a secondary camera. Secondly, the current prototype setup has limited processing time which is around 20 fps whereas the conventional surgical microscope video rate is 60 fps [29]. Low video processing time affected the performance of participants by interfering with hand and eye coordination during the task. As noted from the participant feedback, speed lag required time for performers to get acquainted with lower fps compared to their conventional setting. New tests with more powerful hardware, particularly workstation PC can eliminate frame rate decrease and consequently speed lag. Finally, we had a limited number of participants for both interviews (*N* = 16) and prototype testing (*N* = 12) which limits the statistical power of correlations. A larger study sample and prior training are needed to evaluate the impact of a solution.

With increased attention to visual ergonomics in surgery the importance of assessment tools is in demand. Compared to postural ergonomics, which has been extensively studied and incorporated into training, visual ergonomics assessment remains limited. In this study we used a questionnaire to explore the neurosurgeons’ perceptions, but future research should include quantitative and objective evaluation tools such as eye tracking, gaze analysis and head and neck movements[16, 18, 23]. In addition, assessment of new developments should include some evaluation tools and simulation of real surgical scenes e.g. narrow passages.

## Conclusion

We quantified and surveyed types of visual challenges among neurosurgeons and proposed software-based solutions. The solutions, namely context-preserving magnification (CPM) and instrument transparency (IT) showed potential of mitigating these two visual ergonomics challenges.

## Data Availability

Data is available upon a reasonable request.

## References

[1] Afifi A, Takada C, Yoshimura Y, Nakaguchi T (Mar.2021) Real-Time Expanded Field-of-View for Minimally Invasive Surgery Using Multi-Camera Visual Simultaneous Localization and Mapping. Sensors (Basel) 21(6):1–20. 10.3390/S21062106 10.3390/s21062106 PMC8002421 33802766

[2] Alhusuny A, Cook M, Khalil A, Johnston V (Jul.2021) Visual symptoms, Neck/shoulder problems and associated factors among surgeons performing Minimally Invasive Surgeries (MIS): A comprehensive survey. Int Arch Occup Environ Health 94(5):959–979. 10.1007/S00420-020-01642-2/TABLES/3 33515063 10.1007/s00420-020-01642-2

[3] Belykh E (2018) Microvascular anastomosis under 3D exoscope or endoscope magnification: a proof-of-concept study. Surg Neurol Int. 10.4103/sni.sni_36_18 30105125 10.4103/sni.sni_36_18 PMC6070836

[4] Boaro A (2022) Visualization, navigation, augmentation. The ever-changing perspective of the neurosurgeon. Brain & Spine 2:100926. 10.1016/J.BAS.2022.100926 36248169 10.1016/j.bas.2022.100926 PMC9560703

[5] Bogdanova R, Boulanger P, Zheng B (2016) Depth perception of surgeons in minimally invasive surgery. Surg Innov 23(5):515–524. 10.1177/1553350616639141 27009686 10.1177/1553350616639141

[6] Dias RD, Ngo-Howard MC, Boskovski MT, Zenati MA, Yule SJ (2018) Systematic review of measurement tools to assess surgeons’ intraoperative cognitive workload. Br J Surg. 10.1002/bjs.10795 29465749 10.1002/bjs.10795 PMC5878696

[7] Dutton J et al (Apr.2020) Influence of stereopsis on the ability to perform simulated microsurgery. J Cataract Refract Surg 46(4):549–554. 10.1097/J.JCRS.0000000000000090 32271521 10.1097/j.jcrs.0000000000000090

[8] Eivazi S, Afkari H, Bednarik R, Leinonen V, Tukiainen M, Jääskeläinen JE (Jul.2015) Analysis of disruptive events and precarious situations caused by interaction with neurosurgical microscope. Acta Neurochir (Wien) 157(7):1147–1154. 10.1007/S00701-015-2433-5/FIGURES/4 25962996 10.1007/s00701-015-2433-5

[9] El Beltagy MA, Atteya MME (2021) Benefits of endoscope-assisted microsurgery in the management of pediatric brain tumors. Neurosurg Focus 50(1):E7. 10.3171/2020.10.FOCUS20620 33386008 10.3171/2020.10.FOCUS20620

[10] Hatzipanayioti A et al (Oct.2021) Associations Between Binocular Depth Perception and Performance Gains in Laparoscopic Skill Acquisition. Front Hum Neurosci 15:675700. 10.3389/FNHUM.2021.675700/FULL 34675789 10.3389/fnhum.2021.675700 PMC8524002

[11] Kelkar A, Natarajan S, Kothari A, Bolisetty M (2024) Comparison of cognitive workload and surgical outcomes between a three-dimensional and conventional microscope macular hole surgery. BMC Ophthalmol. 10.1186/s12886-024-03361-5 38429711 10.1186/s12886-024-03361-5 PMC10908162

[12] Khakhar R, You F, Chakkalakal D, Dobbelstein D, Picht T (2021) Hands-free adjustment of the microscope in microneurosurgery. World Neurosurg 148:e155–e163. 10.1016/j.wneu.2020.12.092 33385607 10.1016/j.wneu.2020.12.092

[13] Koreeda Y et al (2016) Virtually transparent surgical instruments in endoscopic surgery with augmentation of obscured regions. Int J Comput Assist Radiol Surg 11(10):1927–1936. 10.1007/S11548-016-1384-5 27038964 10.1007/s11548-016-1384-5

[14] Koskinen J (2022) Computational Evaluation of Microsurgical Skills. University of Eastern Finland, Joensuu. http://urn.fi/URN:ISBN:978-952-61-4743-7

[15] Koskinen J, Torkamani-Azar M, Hussein A, Huotarinen A, Bednarik R (2022) Automated tool detection with deep learning for monitoring kinematics and eye-hand coordination in microsurgery. Comput Biol Med. 10.1016/j.compbiomed.2021.105121 34968859 10.1016/j.compbiomed.2021.105121

[16] Lavé A, Gondar R, Demetriades AK, Meling TR (2020) Ergonomics and musculoskeletal disorders in neurosurgery: a systematic review. Acta Neurochir (Wien) 162:2213–2220. 10.1007/s00701-020-04494-4 32705353 10.1007/s00701-020-04494-4 PMC7415019

[17] Lerotic M, Chung AJ, Clark J, Valibeik S, Yang GZ (2008) Dynamic view expansion for enhanced navigation in natural orifice transluminal endoscopic surgery. Lecture Notes in Computer Science (including subseries Lecture Notes in Artificial Intelligence and Lecture Notes in Bioinformatics), vol. 5242 LNCS, no. PART 2, pp. 467–475, 10.1007/978-3-540-85990-1_56 10.1007/978-3-540-85990-1_56 18982638

[18] Mavrovounis G, Meling TR, Lafuente J, Fountas KN, Demetriades AK (2021) Postural ergonomics and work-related musculoskeletal disorders in neurosurgery: lessons from an international survey. Acta Neurochir (Wien) 163:1541–1552. 10.1007/s00701-021-04722-5 33594483 10.1007/s00701-021-04722-5 PMC8116287

[19] Miranda-Luna R, Daul C, Blondel WCPM, Hernandez-Mier Y, Wolf D, Guillemin F (2008) Mosaicing of bladder endoscopic image sequences: distortion calibration and registration algorithm. IEEE Trans Biomed Eng 55(2):541–553. 10.1109/TBME.2007.903520 18269989 10.1109/TBME.2007.903520

[20] Nishiyama K (2017) From exoscope into the next generation. J Korean Neurosurg Soc 60(3):289. 10.3340/JKNS.2017.0202.003 28490154 10.3340/jkns.2017.0202.003 PMC5426447

[21] Okubo T, Nakaguchi T, Hayashi H, Tsumura N (n.d.) Abdominal View Expansion by Retractable Camera.

[22] Park W et al (2024) Computational image analysis of distortion, sharpness, and depth of field in a next-generation hybrid exoscopic and microsurgical operative platform. Front Surg. 10.3389/fsurg.2024.1418679 38983589 10.3389/fsurg.2024.1418679 PMC11231637

[23] Schupper AJ, Hrabarchuk EI, McCarthy L, Hadjipanayis CG (2023) Improving surgeon well-being: ergonomics in neurosurgery. World Neurosurg 175:e1220–e1225. 10.1016/j.wneu.2023.04.102 37427701 10.1016/j.wneu.2023.04.102

[24] Takada C, Suzuki T, Afifi A, Nakaguchi T (2017) Hybrid tracking and matching algorithm for mosaicking multiple surgical views. *Lecture Notes in Computer Science (including subseries Lecture Notes in Artificial Intelligence and Lecture Notes in Bioinformatics)*, vol. 10170 LNCS, pp. 24–35, 10.1007/978-3-319-54057-3_3

[25] Torkamani-Azar M, Lee A, Bednarik R (2022) Methods and measures for mental stress assessment in surgery: a systematic review of 20 years of literature. IEEE J Biomed Health Inform. 10.1109/JBHI.2022.3182869 35696473 10.1109/JBHI.2022.3182869

[26] Wang T, Li H, Pu T, Yang L (2023) Microsurgery robots: applications, design, and development. Sensors. 10.3390/S23208503 37896597 10.3390/s23208503 PMC10611418

[27] White AJ, Nowacki AS, Woodrow SI, Steinmetz MP, Benzil DL (2024) Ergonomics, musculoskeletal disorders, and surgeon gender in spine surgery: a survey of practicing spine surgeons. J Neurosurg Spine 40(4):529–538. 10.3171/2023.11.SPINE23705 38215442 10.3171/2023.11.SPINE23705

[28] Wong SW, Crowe P (2023) Visualisation ergonomics and robotic surgery. J Robot Surg 17:1873–1878. 10.1007/s11701-023-01618-7 37204648 10.1007/s11701-023-01618-7 PMC10492791

[29] Yamashita H, Kobayashi E (2021) Mechanism and design of a novel 8K ultra-high-definition video microscope for microsurgery. Heliyon. 10.1016/j.heliyon.2021.e06244 33665425 10.1016/j.heliyon.2021.e06244 PMC7900699

[30] Zhang Z, Feng Y, Lu X, Yang B, Zhang H, Ma Y (2022) Microvascular anastomosis in a challenging setting using a 4 K three-dimensional exoscope compared with a conventional microscope: an in vivo animal study. Front Surg. 10.3389/fsurg.2022.1021098 36338649 10.3389/fsurg.2022.1021098 PMC9630566

